# Supplementary material for: Quality of life and associated factors among patients with epilepsy at specialized hospitals, Northwest Ethiopia; 2019
Source: PLoS One. 2022 Jan 27;17(1):e0262814. doi: 10.1371/journal.pone.0262814 (PMC8794165; doi:10.1371/journal.pone.0262814)
Supplement: S1 File — (DOCX) [file pone.0262814.s001.docx]

**Part I: Socio-demographic questionnaires**

| S.No | Questions | Response | Skip |
| --- | --- | --- | --- |
| 101 | Sex of the respondents? | 1. Male 2. Female |  |
| 102 | How old are you? | ----------------------years |  |
| 103 | What is your religion | 1. Orthodox Christian 2. Muslim 3. Others----------------- |  |
| 104 | What is your residence area? | 1. Urban 2. Rural |  |
| 105 | Current Marital status? | 1. Single 2. Married  3. Divorced 4. Widowed |  |
| 106 | What is your family size? | ________ in number |  |
| 107 | What is your educational status? | 1. Unable to read and write 2. Able to read and write 3. Primary school 4. Secondary school 5. Diploma and above |  |
| 108 | What is your occupation? | 1. government employee  2. private employee  3. business  4. farmer  5. others(specified)----------------- |  |
| 109 | what is your average monthly income | ____________ Ethiopian Birr |  |

**Part II: clinical factors questionnaire**

| S.No | Questions | Response | Skip |
| --- | --- | --- | --- |
| 201 | How long you have lived with Illness? | 1.------------------month  2.------------------year |  |
| 202 | How many times does seizure occur? | 1. ------------month 2. -----------year 3. Seizure free |  |
| 203 | How long you have taken the drugs? | 1. ------------- months 2. -------------years |  |
| 204 | How many types of drug you have taken? | 1. One 2. Two & above |  |
| 205 | Have you ever had encounter side effect of drug (AED)? | 1. Yes 2. No | If yes skip question 206 |
| 206 | If you say yes to question number 205 which AED more than one answer is possible? | 1. Dizziness 2. Nausea 3. Head ache 4. Tiredness 5. Others(sp ------------- |  |

**Part III. Drug adherence questionnaire**

| S.NO. | QUESTIONS | RESPONSE | |
| --- | --- | --- | --- |
|  |  | YES | NO |
| 301 | Do you sometimes forget to take your pills? | 1 | 0 |
| 302 | People sometimes miss taking their medications for reasons other than forgetting. Thinking over the past two weeks, were there any days when you did not take your medicine? | 1 | 0 |
| 303 | Have you ever cut back or stopped taking your medicine without telling your doctor because you felt worse when you took it? | 1 | 0 |
| 304 | When you travel or leave home, do you sometimes forget to bring along your medicine? | 1 | 0 |
| 305 | Did you take all your medicine yesterday? | 1 | 0 |
| 306 | When you feel like your symptoms are under control, do you sometimes stop taking your medicine? | 1 | 0 |
| 307 | Taking medicine every day is a real inconvenience for some people. Do you ever feel hassled about sticking to your treatment plan? | 1 | 0 |
| 308 | How often do you have difficulty remembering to take all your medicine? | 1.Never/rarely  2. Once in a while  3. Sometimes  4. Usually  5. All the time | |

**Part IV: comorbid health anxiety and depression questionnaire**

Tick the box beside the reply that is closest to how you have been feeling in the past week.

Don’t take too long over your replies: your immediate is best.

| **S.No** | **Questions** | **Response** | |
| --- | --- | --- | --- |
| 401 | I feel tense or 'wound up | Most of the time | 3 |
|  |  | A lot of the time | 2 |
|  |  | Occasionally | 1 |
|  |  | Not at all | 0 |
| 402 | I get a sort of frightened feeling as if  something awful is about to happen: | Very definitely and quite badly | 3 |
|  |  | Yes, but not too badly | 2 |
|  |  | A little, but it doesn't worry me | 1 |
|  |  | Not at all | 0 |
| 403 | Worrying thoughts go through my  Mind | A great deal of the time | 3 |
|  |  | A lot of the time | 2 |
|  |  | From time to time, but not too often | 1 |
|  |  | Only occasionally | 0 |
| 404 | I can sit at ease and feel relaxed | Definitely | 0 |
|  |  | Usually | 1 |
|  |  | Not often | 2 |
|  |  | Not at all | 3 |
| 405 | I get a sort of frightened feeling like ‘butterflies' in the stomach: | Not at all | 0 |
|  |  | Occasionally | 1 |
|  |  | Quite Often | 2 |
|  |  | Very Often | 3 |
| 406 | I feel restless as I have to be on the move | Very much indeed | 3 |
|  |  | Quite a lot | 2 |
|  |  | Not very much | 1 |
|  |  | Not at all | 0 |
| 407 | I get sudden feelings of panic | Very often indeed | 3 |
|  |  | Quite often | 2 |
|  |  | Not very often | 1 |
|  |  | Not at all | 0 |
| 408 | I still enjoy the things I used to  enjoy: | Definitely as much | 0 |
|  |  | Not quite so much | 1 |
|  |  | Only a little | 2 |
|  |  | Hardly at all | 3 |
| 409 | I can laugh and see the funny side  of things: | As much as I always could | 0 |
|  |  | Not quite so much now | 1 |
|  |  | Definitely not so much now | 2 |
|  |  | Not at all | 3 |
| 410 | I feel cheerful | Not at all | 3 |
|  |  | Not often | 2 |
|  |  | Sometimes | 1 |
|  |  | Most of the time | 0 |
| 411 | I feel as if I am slowed down | Nearly all the time | 3 |
|  |  | Very often | 2 |
|  |  | Sometimes | 1 |
|  |  | No at all | 0 |
| 412 | I have lost interest in my appearance: | Definitely | 3 |
|  |  | I don't take as much care as I should | 2 |
|  |  | I may not take quite as much care | 1 |
|  |  | I take just as much care as ever | 0 |
| 413 | I look forward with enjoyment to things: | As much as I ever did | 0 |
|  |  | Rather less than I used to | 1 |
|  |  | Definitely less than I used to | 2 |
|  |  | Hardly at all | 3 |
| 414 | I can enjoy a good book or radio or TV program: | Often | 0 |
|  |  | Sometimes | 1 |
|  |  | Not often | 2 |
|  |  | Very seldom | 3 |

**Part V: stigma scale questionnaires**

Please tell me how much you exactly feel with each of the following 15 questions by encircle the exact answer you fit.

| S.No | Questions | Response scores | | |
| --- | --- | --- | --- | --- |
|  |  | No at all | Sometimes | Always |
| 501 | Do you feel different from other people? | 0 | 1 | 2 |
| 502 | Do you feel lonely? | 0 | 1 | 2 |
| 503 | Do you feel embarrassed? | 0 | 1 | 2 |
| 504 | Do you feel disappointed in yourself? | 0 | 1 | 2 |
| 505 | Do you feel that you cannot have a rewarding life? | 0 | 1 | 2 |
| 506 | Do you feel that you cannot contribute anything in society? | 0 | 1 | 2 |
| 507 | Do you feel that you cannot join others in in public places? | 0 | 1 | 2 |
| 508 | Do you feel that other people are uncomfortable with you? | 0 | 1 | 2 |
| 509 | Do you feel that other people do not want to go to occasions with you? | 0 | 1 | 2 |
| 510 | Do you feel that other people treat you like inferior person? | 0 | 1 | 2 |
| 511 | Do you feel that other people would prefer to avoid you? | 0 | 1 | 2 |
| 512 | Do you feel that other people avoid exchanging greeting with you? | 0 | 1 | 2 |
| 513 | Do you feel that you are mistreated by other people? | 0 | 1 | 2 |
| 514 | Do you feel that other people discriminate against you? | 0 | 1 | 2 |
| 515 | Do you feel that other people treat you like an outcast? | 0 | 1 | 2 |

**Part VI: Quality of life domains questions**

| **s.no** | **Questions** | **Response** | | | | | | |
| --- | --- | --- | --- | --- | --- | --- | --- | --- |
| 601 | \|  \|  \|  \| \| --- \| --- \| --- \|   How would you rate Your quality of life? | Very poor  1 | Poor  2 | | Neutral  3 | Good  4 | | Very good  5 |
| 602 | How satisfied are you with your health? | Very dissatisfied  1 | Dissatisfied  2 | | Neutral  3 | Satisfied  4 | | Very satisfied  5 |
| The following questions ask about **how much** you have experienced certain things in the last times | | | | | | | | |
|  |  | *(Please circle the number)* | | | | | | |
|  |  | Not at all | A little | A moderate amount | | | Very much | An extreme amount |
| 603 | To what extent do you feel that physical pain prevents you from doing what you need to do? | 1 | 2 | 3 | | | 4 | 5 |
| 604 | How much do you need any medical treatment to function in your life? | 1 | 2 | 3 | | | 4 | 5 |
| **605** | How much do you enjoy life? | 1 | 2 | 3 | | | 4 | 5 |
| **606** | To what extent do you feel your life to be meaningful? | 1 | 2 | 3 | | | 4 | 5 |
| **607** | How well are you able to concentrate? | 1 | 2 | 3 | | | 4 | 5 |
| **608** | How safe do you feel in your daily life? | 1 | 2 | 3 | | | 4 | 5 |
| **609** | How healthy is your physical environment? | 1 | 2 | 3 | | | 4 | 5 |
| The following questions ask about **how completely** you experience or were able to do certain things in the last times. | | | | | | | | |
|  |  | *(Please circle the number)* | | | | | | |
|  |  | No at all | A little | Moderate | | | Mostly | Completely |
| **610** | Do you have enough energy  for everyday life? | 1 | 2 | 3 | | | 4 | 5 |
| **611** | Are you able to accept your bodily appearance? | 1 | 2 | 3 | | | 4 | 5 |
| **612** | Have you enough money to meet your needs? | 1 | 2 | 3 | | | 4 | 5 |
| **613** | How available to you is the information that you need in your day-to-day life? | 1 | 2 | 3 | | | 4 | 5 |
| **614** | To what extent do you have the opportunity for leisure activities? | 1 | 2 | 3 | | | 4 | 5 |
| **615** | How well are you able to get around? | Very poor  1 | Poor  2 | Neutral  3 | | | Well  4 | Very well  5 |
| The following questions ask you to say how **good** or **satisfied** you have felt about various aspects of your life over the last times. | | | | | | | | |
|  |  | *(Please circle the number)* | | | | | | |
|  |  | Very dissatisfied | Dissatisfied | Neutral | | | Satisfied | Very satisfied |
| **616** | How satisfied are you with your sleep? | 1 | 2 | 3 | | | 4 | 5 |
| **517** | How satisfied are you with your ability to perform your daily living activities. | 1 | 2 | 3 | | | 4 | 5 |
| **618** | How satisfied are you with your capacity for work? | 1 | 2 | 3 | | | 4 | 5 |
| **619** | How satisfied are you with yourself? | 1 | 2 | 3 | | | 4 | 5 |
| **620** | How satisfied are you with your personal relationships? | 1 | 2 | 3 | | | 4 | 5 |
| **621** | How satisfied are you with your sex life? | 1 | 2 | 3 | | | 4 | 5 |
| **622** | How satisfied are you with the support you get from your friends? | 1 | 2 | 3 | | | 4 | 5 |
| **623** | How satisfied are you with the conditions of your living place? | 1 | 2 | 3 | | | 4 | 5 |
| **624** | How satisfied are you with your access to health services? | 1 | 2 | 3 | | | 4 | 5 |
| **625** | How satisfied are you with your mode of transportation? | 1 | 2 | 3 | | | 4 | 5 |
| The following question refers to **how often** you have felt or experienced certain things in the last times. | | | | | | | | |
| **626** | How often do you have negative feelings, such as blue mood, despair, anxiety, depression? | Never | Seldom | Quit often | | | Very often | Always |
|  |  | 1 | 2 | 3 | | | 4 | 5 |
