# Supplementary material for: Quality of life and associated factors among patients with epilepsy at specialized hospitals, Northwest Ethiopia; 2019
Source: PLoS One. 2022 Jan 27;17(1):e0262814. doi: 10.1371/journal.pone.0262814 (PMC8794165; doi:10.1371/journal.pone.0262814)
Supplement: Attachment — Submitted filename: Review Comments for QoL Ethiopia Manuscript.docx [file pone.0262814.s003.docx]

**Comments**

This is a valuable piece of work that examine quality of life for epilepsy patients using WHO HRQoL-BERF. To determine associated factors of HRQoL authors also considered some clinical factors (adherence, anxiety, and depression) and psychological factor like perceived stigma that also measured using some well-known established scales. It is a very good combination for data base research using different scale for epilepsy patient.

However, substantial improvement is required especially in introduction and methodology section. Some suggestions to improve the manuscript are as follows:

**Overall comments**

1. What is the relevance of this study as of few similar studies published in recent year on the same patients in the same region? More recently (in 2021) published two papers and one of them in PLOSONE as well (DOI: <https://doi.org/10.1371/journal.pone.0247336>).
2. No clear literature gap stated in the introduction section. Major revision is required to update on literature review specially to find the current status of Ethiopia to find out the existing literature gap.
3. What is the relevance to use of HRQOL-BERF to measure QOL for epilepsy patients, although there existing another tool (Quality of Life in Epilepsy Inventory (QOLIE-31)) to measure QOL especially for epilepsy patients.
4. It is required to take permission from developer to use developed tools for research purpose. No statement found about to take permission to use different tools (like: MMAS-8, KESS-15, DAS-14, WHOQOL-BERF-26) for the current study.
5. IRB statement should be stated with the IRB number for more transparency.

**Specific comments:**

**Introduction:**

1. Required to review recent publication
2. Significance of the study and literature/knowledge gap should be stated clearly and decent way as huge lacking found here.

**Methodology:**

1. According to the statement (The total number of epilepsy patients from the two referral hospitals was 1,395, in Debre Markos referral hospital 864 and Shegaw Motta district hospital 531) population size of this study was known. So it is possible to calculate sample size from this known population. Although the current procedure is fine but better to go for strait forward method.
2. Procedure of systematic random sampling should be explain in details
3. Please give the reference to use P-value ≤ 0.2 to select covariates for multiple model. Page 10 L-187-188, In bivariable logistic regression analysis variables having P-value ≤ 0.2 was a potential candidate for multivariable logistic regression analysis.
4. Required to explain properly about the Outcome variable. How classified QOL good vs poor? What is the cutoff value? Have any reference?
5. Similarly explanation also required about SCALING, SCORING and CLASSIFICATION with cutoff point and reference/logic for independent variables ADHERENCE, STIGMA, ANXIETY and DEPRESSION.

**Results:**

1. In table 5 better to report p-value for odds to avoid confliction as higher p-value was used to select variables for multiple regression
2. Very high odds observed for ADHERENCE categories from table 5. Required to crosscheck it.
3. Better to report the result about model fitness under the respective tables

**Discussion:**

1. ‘Results with statistics’ were repeated in the several parts of the discussion section with the results section. Repetition should be prohibited due to redundancy
2. Major revision is required on the writing style as the writing of the manuscript failed to follow scientific merit due to unnecessarily describe some points with repetition of results that increased the volume

**Reference:**

1. Reference is required to update. Reference number 3, 9, 29 updated reference is available.
