# Supplementary material for: Quality of life and associated factors among patients with epilepsy at specialized hospitals, Northwest Ethiopia; 2019
Source: PLoS One. 2022 Jan 27;17(1):e0262814. doi: 10.1371/journal.pone.0262814 (PMC8794165; doi:10.1371/journal.pone.0262814)
Supplement: Attachment — Submitted filename: Point by point response.docx [file pone.0262814.s004.docx]

**Author’s Point-by-Point Response to the Reviewer's and Editors Reports**

**Title**: **Quality of life and associated factors among patients with epilepsy at specialized hospitals, Northwest Ethiopia; 2019**

**Corresponding author**: Asmare Getie/ asmaregetie2017@gmail.com

PONE-D-21-32281

Journal: PLOS ONE
**Point by point response to Reviewers and Editors**

First of all, the authors would like to thank PLOSE ONE Journal editors and the respective reviewers for reviewing this manuscript and providing the necessary comments to be corrected. As per the comments given, we have made modifications and presented point by point to each comment. The authors tried to answer all the issues raised by editorial team and reviewers. Please note that we gave the response in blue font color.

**Reviewer 1: comments and the response given from the authors.**

Comment 1: Results, Would have been interesting to see a breakdown of the results based on institutions since the study was institution based. Kindly do so if possible to identify specific patterns for the individual hospitals if any. Nevertheless, the Results are still interesting and well done.

Response 1: thank you very much for this suggestion. The two institution are located in same region and same zone, the authors believed that there would not be different patterns in this two hospitals. The institution is not considered as a factor to affect the quality of life of patients with epilepsy. That is why the authors didn’t showed the results as breakdown.

General comments and their response: all the comments given in each line by line as well as the grammar part and way of writing style was modified and corrected accordingly. The authors kindly request the reviewer to appreciate the modification from the track change and corrected manuscript.

**Reviewer 2: comments and the response given from the authors.**

Comment 1: What is the relevance of this study as of few similar studies published in recent year on the same patients in the same region? More recently (in 2021) published two papers and one of them in PLOSONE as well (DOI: <https://doi.org/10.1371/journal.pone.0247336>).

Response 1: thank you for your suggestion: even if the two studies was conducted in one region, but they were conducted in different zones. The current study was conducted in East Gojjam Amhara region and that one was conducted in North wolo Amhara region. As we have tried to see the gab, this epileptic patients are highly marginalized and it is very sensitive issue especially in developing countries including Ethiopia. There for further community based as well as institutional based study is very crucial. The researchers are strongly believed that the finding of this study is very significant for both the local and international community as well as for those peoples suffered from epilepsy. In addition since epilepsy is very sensitive issue the belief of the individual their perception towards the problem in two study area might be differ.

Comment 2: No clear literature gap stated in the introduction section. Major revision is required to update on literature review specially to find the current status of Ethiopia to find out the existing literature gap.

Response 2: thank you very much for this constructive suggestion, based on your suggestion the introduction part was revised and the existing current status in Ethiopia was addressed. You can kindly check the modification in the clean manuscript and manuscript with track change.

Comment 3: What is the relevance to use of HRQOL-BERF to measure QOL for epilepsy patients, although there existing another tool (Quality of Life in Epilepsy Inventory (QOLIE-31)) to measure QOL especially for epilepsy patients.

Response 3: thank you very much for this nice suggestion. Reliability, content and construct validity testing has been performed on the QOLS and a number of translations have been made throughout different continent of the world. The QOLS is a valid instrument for measuring quality of life across patient groups and cultures and is conceptually distinct from health status or other causal indicators of quality of life and it is very appropriate to use it in the contextualized ways to the population characters of different study areas. Many different researchers have used this standardized, well validated and highly reliable tools. As a researcher we believed that even we have used Quality of Life in Epilepsy Inventory (QOLIE-31)) to measure QOL especially for epilepsy patients.

Comment 4: It is required to take permission from developer to use developed tools for research purpose. No statement found about to take permission to use different tools (like: MMAS-8, KESS-15, DAS-14, WHOQOL-BERF-26) for the current study.
5. IRB statement should be stated with the IRB number for more transparency.

Response 4: thank you very much for this concern. We believed that putting citation for this tool we have used can take over the permission statement, since citation is one way of giving acknowledgment/recognition to the developer of the original work. Citation was putted there.

Comment 5: IRB statement should be stated with the IRB number for more transparency.

Response 5: thank you very much, it was corrected accordingly HSC/1024/16/11

Comment 6: 1. Required to update with recent publication

Response 6: thank you, it was modified accordingly.
Comment 7: Significance of the study and literature/knowledge gap should be stated clearly and decent way as huge lacking found here.

Response 7: thank you very much for this suggestion, based on this suggestion modification was made and the researchers have tried to address the significance of the study and knowledge gap.

Comment 8: According to the statement (The total number of epilepsy patients from the two referral hospitals was 1,395, in Debre Markos referral hospital 864 and Shegaw Motta district hospital 531) population size of this study was known. So it is possible to calculate sample size from this known population. Although the current procedure is fine but better to go for strait forward method.

Response 8: thank you very much, as you have said we might calculate strait forward, but already we have used the presented procedure.

Comment 9: Procedure of systematic random sampling should be explain in details

Response 9: thank you for this suggestion, the procedure was presented in detail, you are kindly appreciate from the modified manuscript or from the track change.
Comment 10. Please give the reference to use P-value ≤ 0.2 to select covariates for multiple model.

Response 10: thank you very much, actually we have used P-value ≤ 0.25 to select covariates for multiple model, it was a type error, and was corrected accordingly.

Comment 11: Required to explain properly about the Outcome variable. How classified QOL good vs poor? What is the cutoff value? Have any reference?
5. Similarly explanation also required about SCALING, SCORING and CLASSIFICATION with cutoff point and reference/logic for independent variables ADHERENCE, STIGMA, ANXIETY and DEPRESSION.

Response 11: to categorize the outcome variable we have used mean. The mean score of each domain and the total score were also calculated since quality of life measures in studies are often presented as means. Therefore, categorization was done using the mean scores of WHOQOL-BREF. Those respondents who scored greater than or equal to mean were categorized as having GOOD QOL in WHOQOL-BREF, and those subjects with values less than the mean, were categorized as having POOR QOL

We have used Health anxiety and depression scale which was a 14-item questionnaire, commonly used to screen for symptoms of anxiety and depression. The 14- items can be separated into two 7-item sub-scales for each anxiety and depression from 0(zero) to 3(three) likert scale. HADS scale was validated in Ethiopia. The scales was used a cut -off score for anxiety and depression of greater than or equal to 8. Those who had scored 8 and greater than 8 has to be categorized to have anxiety and depression with negative scoring method, the higher the score the more have anxiety and depressed. We have used Kilifi epilepsy stigma scale in Kenya score of 15 to asses Stigma and which was validated in Ethiopia with simple three point Likert scale scoring of” not at all”(0), “sometimes”(1) and “always”(2) and we have used 66^th^ percentile as cut of point to say stigma or not stigma.

To asses drug adherence we have used Morisky Medication Adherence Scales (MMAS-8) which was already used in Ethiopia and it was categorized based on that cutoff points.

Comment 12: Very high odds observed for ADHERENCE categories from table 5. Required to crosscheck it.

Response 12: thank you very much, we have checked the table as well as the model and the output is same. This might indicated that adherence is highly significant variable.

Comment 13: Better to report the result about model fitness under the respective tables

Response 13: thank you for this constructive suggestion, a report about model fitness was incorporated on the manuscript.

Comment 14: ‘Results with statistics’ were repeated in the several parts of the discussion section with the results section. Repetition should be prohibited due to redundancy

Response 14: thank you very much for this critical suggestion, based on the comment we have made a great revision and redundancy was removed. You can kindly appreciate this modification either from the track change or the modified manuscript.

Comment 15: Major revision is required on the writing style as the writing of the manuscript failed to follow scientific merit due to unnecessarily describe some points with repetition of results that increased the volume

Response 15: thank you very much for this constructive comment, based on the suggestion provided we have made a major revision and the writing style as well as the language flow was modified accordingly. You can kindly appreciate this modification either from the track change or the modified manuscript.

Comment 16: Reference is required to update. Reference number 3, 9, 29 updated reference is available.

Response 16: thank you, based on your suggestion given the reference was updated
